# On the Occurrence of Serous Pleurisy Due to Pneumococci and Tubercle Bacilli

**Published:** 1913-06

**Authors:** F. H. Edgeworth

**Affiliations:** Physician to the Bristol Royal Infirmary and Professor of Medicine, University of Bristol.


### ON THE OCCURRENCE OF SEROUS PLEURISY DUE TO PNEUMOCOCCI AND TUBERCLE BACILLI.

BY

F. H. Edgeworth, M.D. Cantab., D.Sc. Lond.,

*Physician to the Bristol Royal Infirmary and Professor of Medicine, University of Bristol.*

---

A. B., male, aged 32, was admitted as an in-patient to the Bristol Royal Infirmary on May 2nd, 1910, complaining of pain in the left side of the chest. He was found to have a temperature of 101° F., a pulse-rate of 84, and a respiration rate of 34 per minute, and to be coughing up a large quantity of mucoid fluid. On further examination, it was discovered that there was a left-sided pleuritic effusion, so extensive as to displace the heart to the right side, and to make the spleen palpable below the left costal margin. The blood contained 9,860 white cells per c.m., and a differential count showed 73 per cent. polymorphonuclears, 15 per cent. small lymphocytes, 10 per cent. large lymphocytes, 1 per cent. eosinophils, and 1 per cent. basophils. The temperature was found to persist, and the effusion to show no signs of lessening, so on May 4th he was tapped, and 24 oz. of a clear yellow serous fluid withdrawn from the left side of the chest.

This pleuritic fluid contained pneumococcus and staphylococcus albus, the latter probably due to skin contamination. Cytodiagnosis of the pleuritic fluid showed 95 per cent. small lymphocytes, 3 per cent. endothelials, and 2 per cent. polynuclears. Pneumococci were also present in the expectorated mucoid fluid.

Now pleural effusions caused by pneumococci are generally purulent, rarely serous in character. The singularity of the case was heightened by the fact that the cell-count of the pleuritic fluid showed 95 per cent. small lymphocytes. For it has been stated by Widal and Ravaut that " when the dominant cells present in a pleural effusion are small lymphocytes, the probable cause of the effusion is the tubercle bacillus ; when the dominant cells are the polymorphonuclear leucocytes, the probable cause is some pyogenic organism."

It seemed advisable, therefore, to make further investigations. A guinea-pig was inoculated with some of the pleuritic effusion. The report returned was : " The experimental animal, weighing 180 grams, died on June 25th, six weeks after inoculation, having gained 90 grams in weight. Fourteen days before death there was a small local lesion. P.M.—There was a generalised tuberculous infection with tubercle bacilli present in the lesions."

It thus became evident that the serous pleurisy was due to a double infection—by pneumococci and tubercle bacilli, whilst the bronchial secretion was due to pneumococci.

Such a causation of a serous pleural effusion is somewhat exceptional. Thus Gee and Horder (in Allbutt's *System*) state that 16 per cent. of purulent pleuritic effusions are due to multiple infections, but they do not mention the occurrence of multiple infections in the case of serous effusions, which are generally due either to tubercle bacilli, or to pneumococci or to streptococci.

The further history of the case may be briefly recorded. The pleuritic effusion rapidly re-accumulated, necessitating a second aspiration on May 10th, when 96 oz. were removed. After that date slow absorption of the remaining fluid took place. The bronchial secretion and the hectic temperature gradually subsided in the course of four weeks, and the patient left the Infirmary on June 25th apparently quite well. He was advised to change his occupation—of a house painter—to one of work on a farm or garden.

